# Increased cancer awareness among British adolescents after a school-based educational intervention: a controlled before-and-after study with 6-month follow-up

**DOI:** 10.1186/1471-2458-13-190

**Published:** 2013-03-04

**Authors:** Richard G Kyle, Liz Forbat, Petra Rauchhaus, Gill Hubbard

**Affiliations:** 1 Cancer Care Research Centre, School of Nursing, Midwifery and Health, University of Stirling, FK9 4LA, Stirling, UK; 2 College of Medicine, Dentistry and Nursing, University of Dundee, DD1 5EH, Dundee, UK

## Abstract

**Background:**

There is a lack of evidence around the effectiveness of school-based interventions designed to raise adolescents’ cancer awareness. To address this deficit this study assessed the impact of an intervention delivered in the United Kingdom by Teenage Cancer Trust on: recall (open question) and recognition (closed question) of cancer warning signs; knowledge of common childhood, teenage, male and female cancers; awareness of the relationship between cancer and age; anticipated medical help-seeking delay; perceived barriers to seeking medical advice about cancer; and examined variation of intervention effect by gender and whether adolescents reported that they knew someone with cancer.

**Methods:**

The Cancer Awareness Measure (CAM) was completed by 422 adolescents (male: 221, 52.4%) aged 11-17 years old (mean age=13.8, standard deviation=1.26) two weeks before and two weeks after the intervention in three schools, and on two occasions four weeks apart in a fourth (control) school. Intervention schools were followed-up 6-months post-intervention.

**Results:**

Recognition of nine common cancer warning signs significantly increased two weeks after the intervention (4.6 to 6.8, p<0.001) and was maintained at 6-month follow-up (6.2, p<0.001). Endorsement of emotional barriers to help-seeking ‘not confident to talk about symptoms’ (53% to 45%, p=0.021) and ‘worried about what the doctor might find’ (70% to 63%, p=0.021) significantly decreased two weeks after the intervention but changes were not maintained at 6-months. The intervention had a greater impact on females and those who knew someone with cancer.

**Conclusions:**

The intervention is an effective way to raise adolescents’ cancer awareness, especially of cancer symptoms. Further development and evaluation is required to maximise intervention impact, particularly on barriers to help-seeking behaviour.

## Background

In the UK around 2,000 new diagnoses of cancer are made each year in teenagers and young adults (TYA) aged 15-24 years, which constitutes 0.6% of all cancer registrations [1]. Malignant melanoma is the most common cancer among female TYAs (17% of all female TYA cancer registrations) and testicular cancer is the most common among male TYAs (27%) [1].

Detecting cancer early reduces the risk of dying from some cancers [2,3]. Indeed, through the National Awareness and Early Diagnosis Initiative (NAEDI) in England and Detect Cancer Early in Scotland, UK government health departments are committed to improving public cancer awareness, recognising it as one component of a comprehensive strategy to increase the proportion of people with early diagnosis [4,5]. Late detection is multifactorial, but patient delay in visiting a General Practitioner (GP) may partially explain the problem [6-8] and lack of public awareness of cancer signs and symptoms may be further reasons for late diagnoses [9].

Adolescents can be easily reached through schools and are thereby a useful arena for raising adolescent cancer awareness. Schools are established loci for health promotion. Examples include the World Health Organisation Global School Health Initiative [10] and Schools for Health in Europe Network, which are designed to instil school-wide health promotion and education activities in policy and practice by changing school health policies, physical environment, community relationships, personal health skills and health services [11]. Schools vary in choice of health topics addressed and strategies adopted [12-17] but there are few school-based programmes designed to raise cancer awareness and none that have been evaluated [18].

Literature about the effectiveness of school-based health interventions may provide useful pointers for developing cancer awareness programmes. Smoking is one of the most researched adolescent health behaviours. Yet, even for smoking, no firm conclusions can be drawn about what are the most effective school-based interventions to raise awareness of the harmful effects of smoking and prevent smoking uptake [19]. A Cochrane review of 76 randomised controlled trials (RCT) of school-based interventions for preventing smoking highlighted conflicting evidence and lack of consensus about the effectiveness of different types of school-based interventions [19]. Smoking is, however, an addictive behaviour and therefore may require a different type of intervention compared to raising adolescent cancer awareness because core determinants and moderating factors for addictive and non-addictive behaviours are likely to vary.

The most well documented field of health promotion in adolescents related to (physically) non-addictive behaviour is around sun awareness and sun protection. A review of 84 interventions, 25 of which (30%) were delivered to TYAs in educational settings including universities (n=18) and secondary schools (n=7), concluded that it was not possible to determine what content or component of an intervention was the most effective [20]. The review included four RCTs involving combinations of video and print material in secondary schools (children aged 12 to 16) and reported that all studies found significant increases in self-reported knowledge of skin cancer risk, measured by questionnaire, between the intervention and control groups between 1 week and 3 months post-intervention [20]. The review also reported that one before-and-after study found significant increases in attitudes towards protecting children from the sun among females only and that another before-and-after study found no significant difference in reported use of sunscreen, hats or sunglasses at 5 month follow-up [20]. Given limitations in methodology, however, the author suggests that it is unlikely that any of these interventions have applicability beyond the population and setting studied.

This paper reports findings of a controlled before-and-after study of a secondary school-based intervention to raise adolescent cancer awareness that was conducted in Scotland and England in 2011. The aim of the study was to address lack of evidence about the effectiveness of school-based interventions. Study objectives were to test if the intervention impacted on the following outcomes: recall (open question) and recognition (closed question) of cancer warning signs; knowledge of common childhood, teenage, male and female cancers; awareness of the relationship between cancer and age; anticipated medical help-seeking delay; perceived barriers to seeking medical advice about cancer; and examined variation of intervention effect by gender and whether adolescents reported that they knew someone with cancer.

## Methods

### Data collection

Data were collected from adolescents aged between 11 and 17 years old, recruited from four schools in Scotland and England between August and October 2011. Schools with an existing relationship with Teenage Cancer Trust were purposively sampled to maximise geographic and age distribution and to ensure both male and female adolescents were included in the study (i.e., single-sex schools were excluded). Thus the sampling strategy incorporated elements of both convenience and purposive sampling. Data were collected during a single day on two occasions in the control school, and three times in the three intervention schools. Individuals who were absent from school (e.g., due to illness) on data collection days were therefore not included in the study because data could not be paired. There were 558 adolescents available to participate in the study (i.e., on the school roll); 422 (75.6%) provided data at baseline and two-week follow-up, and 222 (of 290, 76.6%) students in intervention schools provided six-month follow-up data (Table 1).

**Table 1:** Study response rates

	**School**	**Study participants**				
	**Roll (SR)**	**2-week follow-up (T1)**		**6-month follow-up (T2)**		
**School (English Education System Year)**	**n**	**n**	**% (SR)**	**n**	**% (of SR)**	**% (of T1)**
A (Year 11)	175	124	70.9	104	59.4	83.9
B (Year 10)	174	138	79.3	106	60.9	76.8
C (Year 12)	44	28	63.6	12	27.3	42.9
D (Year 8)	165	132	80.0	-	-	-
Total^†^	558	422	75.6	222	56.5	76.6

Note: ^†^ For 6-month follow-up sub-sample indicates percentage of intervention school total (SR=393, T_1_=290).

### Intervention

The intervention was an educational programme called ‘Let’s talk about it’ delivered by Teenage Cancer Trust in approximately 10% of UK schools each year (n=600). ‘Let’s talk about it’ is a one hour presentation delivered verbally by a single Teenage Cancer Trust educator to groups of adolescents in a classroom or assembly setting. Content is linked to outcomes from the ‘Health and Well-being’ section of the Curriculum for Excellence in Scotland [21] and Personal, Social, Health and Economic Education (PSHEE) in England and Wales [22]. Topics covered in the presentation include: an introduction to cancer; identification of cancer warning signs; the physical, emotional and social impact of cancer; cancer diagnosis and treatment; and the importance of taking responsibility for your own health and well-being.

‘Let’s talk about it’ was delivered in three schools and although the topics covered (i.e., the key messages) did not vary, two out of three presenters showed a DVD (in schools A and B [Table 1]), which includes teenagers talking about their experiences of being diagnosed, treated and living with cancer. All three presenters provided the school with Teenage Cancer Trust student booklets. A fourth (control) school did not receive the intervention.

### Survey instrument

Teachers administered a paper questionnaire to a whole class. Students were asked to complete the questionnaire in complete silence but were informed that it was not a test. Teachers encouraged students to complete as much of the questionnaire as they could. Students were allowed as much time as they needed within the 55 minute lesson, although most completed the questionnaire within 20 minutes. In the three intervention schools the questionnaire was completed two weeks before (T_0_) and again by the same adolescents two weeks (T_1_) and six months (T_2_) after Teenage Cancer Trust delivered the educational intervention. In the control school the questionnaire was completed on two occasions four weeks apart (T_0_ and T_1_). The instrument incorporated the Cancer Awareness Measure (CAM) and socio-demographic questions.

#### Cancer awareness

Cancer awareness was assessed using items from the CAM [23]. Specifically, the study assessed adolescents’: recall (open question) and recognition (closed question) of cancer warning signs; knowledge of common childhood, teenage, male and female cancers; awareness of the relationship between cancer and age; anticipated medical help-seeking delay; and perceived barriers to seeking medical advice about cancer. CAM questions are detailed elsewhere [23] and readers are directed to the tables for further information on specific items. To simplify the instrument and increase its relevance to adolescents two minor changes were made to the CAM in consultation with adolescents from Highland Youth Voice (a youth parliament for the Scottish Highlands [24]) which are described in detail elsewhere [25].

#### Individual-level explanatory variables

Socio-demographic questions were included to gather data on: age, gender, ethnicity (using census categories), and whether the student had been diagnosed with cancer or knew a relative or friend who had been diagnosed with cancer.

### Analysis

Data were analysed using SPSS 19.0. Descriptive statistics were calculated for socio-demographic variables and CAM items. Pearson’s chi-square (*χ*^2^) tests were used to examine differences in socio-demographic characteristics between the intervention and control groups at T_0_. McNemar’s chi-square (χ^2^_M_) tests for matched paired categorical data were used to examine change in recognition of specific cancer warning signs, endorsement of particular barriers to help-seeking, and awareness of common cancers and the relationship between cancer and age within the intervention and control groups between T_0_ and T_1_. Cochran’s Q tests were used to examine change in cancer warning signs recognised, barriers endorsed, and awareness of common cancers and the relationship between cancer and age within the intervention group between T_0_ and T_2_, followed (where appropriate) by planned post-hoc McNemar chi-squared tests with Bonferroni correction. Due to non-normal distributions, Wilcoxon Signed Rank Tests (T) for paired continuous data were used to examine differences in the mean number of cancer warning signs recognised and barriers endorsed between T_0_ and T_1_ in the intervention and control schools. The Friedman test was used to examine differences in the mean number of warning signs recognised and barriers endorsed between T_0_ and T_2_ in the intervention schools followed (where appropriate) by planned Wilcoxon Signed Rank Tests with Bonferroni correction. Change was also examined in the intervention and control groups by gender and whether individuals knew someone with cancer.

### Ethical considerations

The study was reviewed by the Research Ethics Committee in the School of Nursing, Midwifery and Health, University of Stirling which approved the following process for obtaining informed consent from children and young people in the study and their parents/carers. Parents/carers were informed of the study by letter and could opt their child out of the research, but none chose to do so. Written informed consent was obtained from each adolescent before completion of the questionnaire.

## Results

### Sample

The sample included 422 adolescents (male: n=221, 52.4%) aged between 11 and 17 years old (mean age=13.8, Standard Deviation [SD]=1.26). Socio-demographic characteristics of respondents are shown in Table 2.

**Table 2:** Sample demographic characteristics

	**Total (n=422)**		**Intervention (n=290)**		**Control (n=132)**			**6-month follow-up (n=222)**	
	**n**	**%**	**n**	**%**	**n**	**%**	**Significance**^**†**^	**n**	**%**
*Gender*									
Male	221	52.4	162	55.9	59	44.7		127	57.2
Female	201	47.6	128	44.1	73	55.3	p=0.033	95	42.8
*Age*									
11	1	0.2	-	-	1	0.8		-	-
12	104	24.6	-	-	104	78.8		-	-
13	23	5.5	-	-	23	17.4		-	-
14	169	40.0	169	58.3	-	-		137	61.7
15	80	19.0	80	27.6	-	-		64	28.8
16	26	6.2	26	9.0	-	-		11	5.0
17	4	0.9	4	1.4	-	-		2	0.9
Missing	15	3.6	11	3.8	4	3.0	p<0.001	8	3.6
*Ethnicity*									
White	386	91.5	261	90.0	125	94.7		200	90.1
Other ethnic backgrounds	30	7.1	23	7.9	7	5.3		17	7.7
Missing	6	1.4	6	2.1	0	0	p=0.193	5	2.3
*Knew someone with cancer*									
Yes	255	60.4	179	61.7	76	57.6		137	61.7
No	131	31.0	88	30.3	43	32.6		66	29.7
Do not wish to answer	36	8.5	23	7.9	13	9.8	p=0.644	19	8.6
*School (Region)*									
A (Scottish Highlands)	124	29.4	124	42.8	-	-		104	46.8
B (South West England)	138	32.7	138	47.6	-	-		106	47.7
C (English East Midlands)	28	6.6	28	9.7	-	-		12	5.4
D (North West England)	132	31.3	-	-	132	100.0	p<0.001	-	-
*Country*									
Scotland	124	29.4	124	42.8	-	-		104	46.8
England	298	70.6	166	57.2	132	100.0	p<0.001	118	53.2

Note: ^†^ Pearson’s *χ*^2^ test.

### Recall of cancer warning signs

Recall of cancer warning signs improved two weeks after the intervention (Table 3). In the intervention schools there was a statistically significant decrease in the percentage of adolescents who reported that they did not know a cancer warning sign (23.8% to 11.4%, change: -12.4; χ^2^_M_: p<0.001) (Table 3). The greatest improvement in recall was found for ‘unexplained pain’ (an increase of 41.0 percentage points), followed by ‘tiredness/fatigue’ (28.6), and ‘change in the appearance of a mole’ (20.7) (Table 3). There was a significant decrease in the percentage of adolescents who reported ‘hair loss’ as a cancer warning sign (12.4% to 7.2%, change: -5.2; χ^2^_M_: p=0.024). Improved recall of cancer warning signs was maintained at six months for seven symptoms: pain; tiredness/fatigue; change in the appearance of a mole; weight loss; bleeding (Cochran’s Q: all p<0.001; post-hoc χ^2^_M_: all p<0.001); change in bowel/bladder habits (Cochran’s Q: p<0.001; post-hoc χ^2^_M_: p=0.006); bruising (Cochran’s Q: p=0.001; post-hoc χ^2^_M_: p=0.013).

**Table 3:** Recall of cancer warning signs

**Cancer warning sign Yes % (n)**	**Intervention (n=290)**				**Control (n=132)**			
	**T**_**0**_	**T**_**1**_	**Change**	**Significance**^**†**^	**T**_**0**_	**T**_**1**_	**Change**	**Significance**^**†**^
Pain	**13.1 (38)**	**54.1 (157)**	**41.0 (119)**	**p<0.001**	15.2 (20)	16.7 (22)	1.5 (2)	p=0.832
Tiredness/Fatigue	**10.0 (29)**	**38.6 (112)**	**28.6 (83)**	**p<0.001**	2.3 (3)	3.0 (4)	0.7 (1)	p=1.000
Change in appearance of mole	**14.8 (43)**	**35.5 (103)**	**20.7 (60)**	**p<0.001**	12.1 (16)	7.6 (10)	-4.5 (-6)	p=0.180
Weight loss	**5.5 (16)**	**24.8 (72)**	**19.3 (56)**	**p<0.001**	8.3 (11)	5.3 (7)	-3.0 (-4)	p=0.388
Lump	**66.9 (194)**	**82.4 (239)**	**15.5 (45)**	**p<0.001**	62.1 (82)	65.9 (87)	3.8 (5)	p=0.424
Bleeding	**10.0 (29)**	**24.5 (71)**	**14.5 (42)**	**p<0.001**	**4.5 (6)**	**13.6 (18)**	**9.1 (12)**	**p=0.008**
Bowel/Bladder Habits	**1.4 (4)**	**10.3 (30)**	**8.9 (26)**	**p<0.001**	2.3 (3)	5.3 (7)	3.0 (4)	p=0.344
Cough	**8.6 (25)**	**16.6 (48)**	**8.0 (23)**	**p<0.001**	9.8 (13)	16.7 (22)	6.9 (9)	p=0.078
Headache/Migraine	**11.7 (34)**	**19.3 (56)**	**7.6 (22)**	**p=0.003**	**3.8 (5)**	**9.8 (13)**	**6.0 (8)**	**p=0.039**
Stomach ache	**1.4 (4)**	**9.0 (26)**	**7.6 (22)**	**p<0.001**	0 (0)	0.8 (1)	0.8 (1)	^‡^
Weight gain	0 (0)	7.6 (22)	7.6 (22)	^‡^	1.5 (2)	1.5 (2)	0.0 (0)	p=1.000
Sore throat	**0.7 (2)**	**6.6 (19)**	**5.9 (17)**	**p<0.001**	0.8 (1)	3.8 (5)	3.0 (4)	p=0.219
Bruising	**1.4 (4)**	**6.6 (19)**	**5.2 (15)**	**p=0.001**	1.5 (2)	2.3 (3)	0.8 (1)	p=1.000
Nausea/Sickness	**9.7 (28)**	**14.1 (41)**	**4.4 (13)**	**p=0.002**	10.6 (14)	8.3 (11)	-2.3 (-3)	p=0.607
Spots/rashes	2.4 (7)	4.5 (13)	2.1 (6)	p=0.210	3.8 (5)	6.8 (9)	3.0 (4)	p=0.424
Difficulty swallowing	0 (0)	2.1 (6)	2.1 (6)	^‡^	2.3 (3)	1.5 (2)	-0.8 (-1)	p=1.000
Dizziness	1.0 (3)	2.8 (8)	1.8 (5)	p=0.180	3.0 (4)	3.0 (4)	0 (0)	p=1.000
Sore that doesn't heal	1.4 (4)	3.1 (9)	1.7 (5)	p=0.227	1.5 (2)	0.8 (1)	-0.7 (-1)	p=1.000
Loss of appetite	0 (0)	0.7 (2)	0.7 (2)	^‡^	2.3 (3)	3.0 (4)	0.7 (1)	p=1.000
Infection	1.0 (3)	1.0 (3)	0 (0)	p=1.000	0 (0)	0 (0)	0 (0)	^‡^
Cramps	1.0 (3)	0.7 (2)	-0.3 (-1)	p=1.000	0 (0)	1.5 (2)	1.5 (2)	^‡^
Feeling weak	1.7 (5)	1.0 (3)	-0.7 (-2)	p=0.687	2.3 (3)	3.0 (4)	0.7 (1)	p=1.000
Blurred vision	1.0 (3)	0.3 (1)	-0.7 (-2)	p=0.625	**0.8 (1)**	**5.3 (7)**	**4.5 (6)**	**p=0.031**
Flu symptoms	1.7 (5)	0.7 (2)	-1.0 (-3)	p=0.250	0 (0)	0 (0)	0 (0)	^‡^
Tumour/Growth	4.8 (14)	3.4 (10)	-1.4 (-4)	p=0.454	0 (0)	0 (0)	0 (0)	^‡^
Generally unwell	5.5 (16)	3.4 (10)	-2.1 (-6)	p=0.286	9.8 (13)	8.3 (11)	-1.5 (-2)	p=0.804
Breathing problems	**4.1 (12)**	**0.3 (1)**	**-3.8 (-11)**	**p=0.003**	1.5 (2)	4.5 (6)	3.0 (4)	p=0.219
Hair loss	**12.4 (36)**	**7.2 (21)**	**-5.2 (-15)**	**p=0.024**	**18.9 (25)**	**29.5 (39)**	**10.6 (14)**	**p=0.024**
Don't know	**23.8 (69)**	**11.4 (33)**	**-12.4 (-36)**	**p<0.001**	**28.8 (38)**	**18.9 (25)**	**-9.9 (-13)**	**p=0.011**

Notes:

^†^ McNemar’s chi-square test for 2x2 tables (i.e., Yes vs. No). Statistically significant differences at the p<0.05 level are emboldened.

^‡^ McNemar’s chi-square test cannot be calculated.

There were smaller variations in recall of cancer warning signs in the control school between T_0_ and T_1_ (Table 3). There was a statistically significant decrease in the percentage of adolescents who reported that they did not know a cancer warning sign (28.8% to 18.9%, change: -9.9, χ^2^_M_: p=0.011) (Table 3). The greatest change was found for ‘hair loss’ (which is not a cancer warning sign) which increased (18.9% to 29.5%, change: 10.6, χ^2^_M_: p=0.024). Recall of ‘change in the appearance of a mole’ and ‘weight loss’ actually decreased (Table 3).

### Recognition of cancer warning signs

Recognition of all nine common cancer warning signs increased two weeks after the intervention (Table 4). With the exception of ‘lump or swelling’, which was the most recognised symptom before the intervention (93.4%), there was a statistically significant increase in recognition for all cancer warning signs two weeks after the intervention (χ^2^_M_: all p<0.001) (Table 4). The greatest increase in recognition was found for ‘unexplained pain’ (an increase of 37.1 percentage points), followed by ‘unexplained weight loss’ (33.9), and ‘sore that does not heal’ (28.8) (Table 4). Improved recognition of cancer warning signs was maintained at six months for all cancer warning signs (Cochran’s Q: all p<0.001; post-hoc χ^2^_M_: all p<0.001, except ‘difficulty swallowing’, p=0.003).

**Table 4:** Recognition of cancer warning signs

**Cancer warning sign Yes % (n)**	**Intervention (n=290)**				**Control (n=132)**			
	**T**_**0**_	**T**_**1**_	**Change**	**Significance**^**†**^	**T**_**0**_	**T**_**1**_	**Change**	**Significance**^**†**^
Unexplained pain	**51.4 (147)**	**88.5 (253)**	**37.1 (106)**	**p<0.001**	**37.4 (49)**	**48.1 (63)**	**10.7 (14)**	**p=0.024**
Unexplained weight loss	**45.1 (129)**	**79.0 (226)**	**33.9 (97)**	**p<0.001**	**32.8 (43)**	**42.7 (56)**	**9.9 (13)**	**p=0.041**
Sore that does not heal	**28.2 (80)**	**57.0 (162)**	**28.8 (82)**	**p<0.001**	17.4 (23)	23.5 (31)	6.1 (8)	p=0.169
Cough or hoarseness	**39.0 (112)**	**66.6 (191)**	**27.6 (79)**	**p<0.001**	**19.7 (26)**	**31.1 (41)**	**11.4 (15)**	**p=0.024**
Unexplained bleeding	**52.8 (151)**	**78.7 (225)**	**25.9 (74)**	**p<0.001**	40.2 (53)	48.5 (64)	8.3 (11)	p=0.117
Difficulty swallowing	**38.9 (111)**	**60.0 (171)**	**21.1 (60)**	**p<0.001**	32.6 (43)	36.4 (48)	3.8 (5)	p=0.511
Change in bowel/bladder habits	**54.4 (155)**	**74.4 (212)**	**20.0 (57)**	**p<0.001**	56.8 (75)	50.8 (67)	-6.0 (-8)	p=0.256
Change in appearance of a mole	**64.9 (185)**	**84.2 (240)**	**19.3 (55)**	**p<0.001**	49.2 (65)	46.2 (61)	-3.0 (-4)	p=0.585
Lump or swelling	93.4 (268)	94.1 (270)	0.7 (2)	p=0.851	80.2 (105)	84.7 (111)	4.5 (6)	p=0.210

Note: ^†^ McNemar’s chi-square test for 2x2 tables (i.e., Yes vs. No/Don’t know). Statistically significant differences at the p<0.05 level are emboldened.

There were smaller increases in recognition for seven out of nine cancer warning signs in the control school between T_0_ and T_1_, and for three symptoms these increases were significant: ‘unexplained pain’ (χ^2^_M_: p=0.024); ‘cough or hoarseness’ (χ^2^_M_: p=0.024); ‘unexplained weight loss’ (χ^2^_M_: p=0.041) (Table 4).

**Figure 1:**
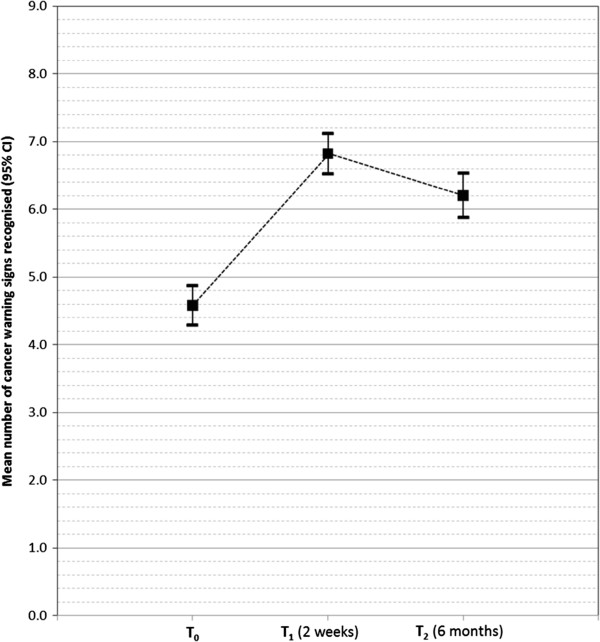
Recognition of cancer warning signs.

Adolescents recognised on average 2.2 more cancer warning signs two weeks after the intervention and this increase was statistically significant (4.6 [SD: 2.21] to 6.8 [SD: 2.26]; T=2247.5, p<0.001). Females showed greater improvement in recognition of cancer warning signs two weeks after the intervention than males (Female: T_0_: 4.7 [SD: 1.98], T_1_: 7.2 [SD: 1.92], change 2.5, T=184.5, p<0.001; Male: T_0_: 4.6 [SD: 2.38], T_1_: 6.4 [SD: 2.44], change 1.8, T=1085.5, p<0.001). There was no difference in improved recognition two weeks after the intervention between adolescents who knew someone with cancer and those who did not (Yes: 4.9 [SD: 1.99] to 7.1 [SD: 2.06], change 2.2, T=839.5, p<0.001; No: 4.2 [SD: 2.48] to 6.3 [SD: 2.46], change 2.2,^1^ T=176.5, p<0.001). Increased recognition of cancer warning signs was maintained at six-months (T_0_: 4.6 [SD=2.20], T_1_: 6.8 [SD=2.23], T_2_: 6.2 [SD=2.44]; Friedman test: *χ*^2^(2, 222)=156.8, p<0.001; post-hoc Wilcoxon Signed Rank Tests: T_0_ to T_2_, p<0.001)) (Figure 1). Increases in recognition six-months post-intervention were greater among females and adolescents who knew someone with cancer (Gender: Female: T_0_: 4.6 [SD=1.94], T_1_: 7.1 [SD=2.06], T_2_: 6.5 [SD=2.22], change 1.9, Friedman test: p<0.001; Male: T_0_: 4.6 [SD=2.38], T_1_:6.6 [SD=2.34], T_2_: 6.0 [SD=2.59], change 1.4, Friedman test: p<0.001; Know someone with cancer: Yes: T_0_: 4.8 [SD=2.00], T_1_: 7.0 [SD=2.17], T_2_: 6.5 [SD=2.36], change 1.7, Friedman test: p<0.001; No: T_0_: 4.2 [SD=2.48], T_1_: 6.5 [SD=2.38], T_2_: 5.6 [SD=2.55], change 1.4, Friedman test: p<0.001).

In the control school adolescents recognised on average 0.4 more cancer warning signs between T_0_ and T_1_ (3.7 [SD: 2.02] to 4.1 [SD: 2.24]; T=1476, p=0.006).

### Awareness of relationship between cancer and age

The percentage of adolescents who believed cancer was unrelated to age significantly decreased two weeks after the intervention (66.9% to 57.5%, change: -9.4; χ^2^_M_: p=0.004); but was not maintained at six months. The percentage of adolescents who believed that someone in their 30s, 40s or 50s was most likely to develop cancer decreased after the intervention whereas the percentage who believed that someone in their 60s, 70s or 80s was most likely to be diagnosed with cancer increased. However, the percentage of adolescents who believed that someone in their 20s was most likely to develop cancer also increased two weeks after the intervention, although this was not statistically significant (4.9% to 6.0%, change: 1.1; χ^2^_M_: p=0.648). Only among females was there a statistically significant increase two weeks after the intervention in the percentage of adolescents who believed someone in their 20s was most likely to develop cancer in the next year (0.8% to 5.7%, change: 4.9; χ^2^_M_: p=0.031); however, this was not evident at six months. There were no significant increases when examined by whether individuals knew someone with cancer.

In the control school there was no statistically significant change in the percentage of adolescents who believed that cancer was unrelated to age (71.3% to 73.0%, change: 1.7; χ^2^_M_: p=0.851).

The decrease in the percentage of adolescents who believed cancer was unrelated to age two weeks after the intervention was greater among females and adolescents who knew someone with cancer than males and those who did not know someone with cancer (Gender: Female: 84.6% to 70.7%, change: -13.9, χ^2^_M_: p=0.002; Male: 51.7% to 46.2%, change: -5.5; χ^2^_M_: p=0.280; Know someone with cancer: Yes: 74.4% to 62.2%, change: -12.2, p=0.003; No: 55.6% to 48.1%, change: -7.5; χ^2^_M_: p=0.286). However, two weeks after the intervention a higher percentage of females and those who knew someone with cancer still believed cancer was unrelated to age compared to males and those who did not know someone with cancer.

### Awareness of common cancers

#### Childhood

The percentage of adolescents who reported that they did not know the most common childhood cancer significantly decreased two weeks after the intervention (Childhood: 49.0% to 26.9%, change: -22.1; χ^2^_M_: p<0.001) and was maintained at six months (Cochran’s Q: p<0.001; post-hoc χ^2^_M_: p=0.001). There was a statistically significant increase in the percentage of adolescents who correctly identified leukaemia as the most common cancer in children two weeks after the intervention (25.9% to 38.3%, change: 12.4; χ^2^_M_: p<0.001) and a significant increase in the percentage of adolescents who thought skin cancer was the most common in childhood (9.0% to 20.3%, change: 11.4, χ^2^_M_: p<0.001); however, these increases were not significant at six months.

In the control school there were no statistically significant changes in the percentage of adolescents reporting that they did not know the most common childhood cancer or identifying leukaemia or skin cancer as the most common.

#### TYA

The percentage of adolescents who reported that they did not know the most common TYA cancer significantly decreased two weeks after the intervention (TYA: 47.9% to 35.5%, change: -12.4; χ^2^_M_: p<0.001); however, this was not significant at six months. There was a statistically significant increase in the percentage of adolescents who identified skin cancer as the most common among TYAs (9.0% to 23.4%, change: 14.5; χ^2^_M_: p<0.001), which also was not maintained at six months, and a small, but not statistically significant, reduction in the percentage of adolescents who reported lung cancer as the commonest TYA cancer (13.4% to 11.0%, change: -2.4; χ^2^_M_: p=0.401).

In the control school there were no statistically significant changes in the percentage of adolescents reporting that they did not know the most common TYA cancer or identifying skin or lung cancer as the most common.

#### Women

There was a surprising statistically significant increase in the percentage of adolescents who reported that they did not know the most common cancer in women (11.4% to 16.2%, change: 4.8; χ^2^_M_: p=0.034) which was also evident at six months (Cochran’s Q: p<0.001; post-hoc χ^2^_M_: p<0.001). There was also a statistically significant decrease in the percentage of adolescents who reported cervical cancer as the most common (6.2% to 2.1%, change: 4.1; χ^2^_M_: p=0.012), although this was not evident at six months. There was no statistically significant change in the percentage of adolescents who correctly identified breast cancer as the most common cancer in women (76.9% to 75.2%, change: -1.7; χ^2^_M_: p=0.615).

In the control school there was no statistically significant change in the percentage of adolescents who reported that they did not know the most common female cancer or identified cervical or breast cancer as the most common.

#### Men

There was a slight, but not statistically significant, decrease two weeks after the intervention in the percentage of adolescents who reported that they did not know the most common male cancer (26.9% to 24.1%, change: -2.8; χ^2^_M_: p=0.382) that was also not significant at six months. There was a statistically significant increase in the percentage of adolescents who reported that testicular cancer was the most common in men (35.9% to 47.6%, change: -11.7; χ^2^_M_: p<0.001); however, this was not evident at six months.

In the control school there was a statistically significant decrease in the percentage of adolescents reporting that they did not know the most common male cancer (53.0% to 39.4%, change: -13.6; χ^2^_M_: p=0.005) and a significant increase in the percentage reporting testicular cancer as the most common in men (19.7% to 30.3%, change: 10.6; χ^2^_M_: p=0.004).

### Anticipated delay

There were no statistically significant changes in the percentage of adolescents who indicated they would seek medical help for a symptom they thought might be cancer within 10 or 3 days at either two weeks or six months post-intervention.

In the control school there were also no statistically significant changes in anticipated delay of >10 days (χ^2^_M_: p=0.210) or >3 days (χ^2^_M_: p=0.134) between T_0_ and T_1_.

### Barriers to help-seeking

Endorsement of seven of the ten barriers to help-seeking, including all four emotional barriers, decreased two weeks after the intervention (Table 5). Statistically significant decreases were found for ‘not confident to talk about symptoms’ (of 8.4 percentage points) and being ‘worried about what the doctor might find’ (7.0; χ^2^_M_: both p=0.021). There was a statistically significant increase in the percentage of adolescents who endorsed the practical barrier ‘other things to worry about’ (22.2% to 29.6%, change: 7.4; χ^2^_M_: p=0.010) (Table 5). However, there were no statistically significant changes in endorsement of any barrier to help-seeking at six months.

**Table 5:** Endorsement of barriers to help-seeking

**Barrier Yes % (n)**	**Intervention (n=290)**				**Control (n=132)**			
	**T**_**0**_	**T**_**1**_	**Change**	**Significance**^**†**^	**T**_**0**_	**T**_**1**_	**Change**	**Significance**^**†**^
**Emotional barriers**								
Not confident to talk about symptoms	**53.0 (151)**	**44.6 (127)**	**-8.4 (-24)**	**p=0.021**	50.0 (65)	43.8 (57)	-6.2 (-8)	p=0.280
Worried about what the doctor might find	**70.2 (200)**	**63.2 (180)**	**-7.0 (-20)**	**p=0.021**	73.5 (97)	70.5 (93)	-3.0 (-4)	p=0.523
Too scared	56.8 (162)	51.9 (148)	-4.9 (-14)	p=0.165	49.6 (65)	43.5 (57)	-6.1 (-8)	p=0.229
Too embarrassed	56.8 (163)	55.7 (160)	-1.1 (-3)	p=0.818	51.5 (67)	52.3 (68)	0.8 (1)	p=1.000
**Practical barriers**								
Other things to worry about	**22.2 (63)**	**29.6 (84)**	**7.4 (21)**	**p=0.010**	12.4 (16)	17.1 (22)	4.7 (6)	p=0.180
Too busy	24.9 (71)	27.7 (79)	2.8 (8)	p=0.389	17.1 (22)	14.7 (19)	-2.4 (-3)	p=0.607
Difficult to arrange transport	16.4 (47)	12.2 (35)	-4.2 (-12)	p=0.111	14.7 (19)	13.2 (17)	-1.5 (-2)	p=0.839
**Service barriers**								
Difficult to make an appointment	23.1 (66)	24.1 (69)	1.0 (3)	p=0.795	15.4 (20)	16.9 (22)	1.5 (2)	p=0.832
Worried about wasting the doctor’s time	34.1 (98)	33.8 (97)	-0.3 (-1)	p=1.000	29.2 (38)	29.2 (38)	0 (0)	p=1.000
Difficult to talk to doctor	35.0 (100)	31.5 (90)	-3.5 (-10)	p=0.282	34.6 (45)	31.5 (41)	-3.1 (-4)	p=0.618

Note: ^†^ McNemar’s chi-square test for 2x2 tables (i.e., Yes vs. No/Don’t know). Statistically significant differences at the p<0.05 level are emboldened.

In the control school there were no statistically significant changes in the percentage of adolescents who endorsed any of the ten barriers between T_0_ and T_1_ (Table 5).

Adolescents endorsed on average 0.2 fewer barriers to help-seeking two weeks after the intervention, although this decrease was not statistically significant (T_0_: 3.9 [SD: 2.37], T_1_: 3.7 [SD: 2.64]; T=12299.5, p=0.273). Females and adolescents who knew someone with cancer showed greater reduction in the mean number of barriers to help-seeking endorsed two weeks after the intervention than males and adolescents who did not know someone with cancer, respectively, although these changes were not statistically significant (Gender: Female: 4.7 [SD: 2.10] to 4.4 [SD: 2.28], change -0.3; T=2232.5, p=0.053; Male: 3.3 [SD: 2.38] to 3.2 [SD: 2.79], change -0.1; T=3866, p=0.859; Knew someone with cancer: Yes: 3.9 [SD: 2.25] to T_1_: 3.7 [SD: 2.55], change -0.2; T=4765.5, p=0.519; No: 3.7 [SD: 2.43] to T_1_: 3.6 [SD: 2.74], change -0.1; T=1312.5, p=0.993). There were also no statistically significant decreases in the mean number of barriers to help-seeking endorsed at six months.

In the control school adolescents also endorsed on average 0.2 fewer cancer warning signs between T_0_ and T_1_ (3.5 [SD=2.05] to 3.3 [SD=2.30]; T=1863.5, p=0.205).

## Discussion

### Intervention effectiveness

This study shows that the presentation delivered by Teenage Cancer Trust raised adolescent cancer awareness. Two weeks post-intervention the presentation significantly increased recall and recognition of cancer warning signs, decreased the proportion of adolescents who reported they did not know the most common cancer among children and TYAs and who believed that cancer was unrelated to age, and challenged some of the common misconceptions about cancer, compared to the fourth school which did not receive the intervention. Moreover, increased cancer awareness was maintained six-months post-intervention for recall and recognition of cancer warning signs.

There was a smaller and less statistically significant increase in recognition of cancer warning signs in the control school compared to the three intervention schools. This suggests that the process of conducting research about cancer may slightly raise adolescents’ cancer awareness. It may be that increased recognition in the control school resulted from adolescents’ conversations with peers, teachers or family members, or information-seeking via the internet, after completion of the initial questionnaire. However, such information-seeking may not address cancer misconceptions. It is notable that in the control school despite a statistically significant decrease in the percentage of adolescents who were unable to recall a cancer warning sign between T_0_ and T_1_ there was a statistically significant increase in the percentage identifying ‘hair loss’ as a cancer warning sign, and decreases in the percentage recalling ‘change in the appearance of a mole’ and ‘weight loss’. Thus, in comparison to the control school the intervention had a consistently positive impact across several dimensions of cancer awareness.

The intervention had a consistently greater impact on females and adolescents who already knew someone with cancer. Other school-based health promotion interventions have also highlighted gender as an explanatory variable including the impact on mental well-being [17]. Thus, future research into adolescents’ cancer awareness should examine gender and knowing someone with cancer as individual-level explanatory variables.

This study therefore lends support for the further development and testing of this school-based intervention to raise adolescent cancer awareness. In particular, the intervention should be developed to address awareness of common male cancers, as well as TYA cancers and the relationship between cancer and age, both of which showed significant increases two weeks post-intervention which decayed by six-month follow-up.

Moreover, these findings suggest that interventions that aim to overcome adolescents’ emotional concerns around visiting their GP should be further developed and evaluated. This intervention development is particularly important as our study also indicated that the intervention did not significantly decrease adolescents’ anticipated delay. Although there is evidence around reasons for delayed cancer diagnosis and clinically useful theoretical models of delay have been developed [6,8] these are adult-based; there is little evidence around reasons for delay in TYAs and no models of adolescent delay. Models of adolescent delay should be developed which acknowledge the importance of emotional barriers to help-seeking.

### Intervention delivery

The study highlights several characteristics of the intervention that may be important for raising cancer awareness. Firstly, the intervention was delivered face-to-face in verbal format and two out of the three presenters also showed a DVD. Teenage Cancer Trust student booklets were also available at the end of the presentation but it is not clear how many students received them. Thus, in the three schools where the presentation was delivered verbal, visual and written information about cancer was effective although it is not possible to determine which method or combination of methods (verbal, visual or written) was most effective. The inability to conclude which method has the greatest impact on health knowledge and behaviours is a weakness of the majority of school-based health promotion research [19,20]. A review of 59 studies (including 26 RCTs) of the effectiveness of educational interventions (irrespective of age group) to increase sun protection knowledge and promote sun protection attitudes and behaviour, for instance, was not able to conclude whether one form of educational intervention (e.g., a leaflet) was better than any other and recommended that future studies should concentrate on comparing different educational interventions to identify which methods are most effective [26]. Moreover, there is limited evidence around the optimum duration of interventions, particularly those delivered face-to-face, to maximise intervention impact. This weakness in the evidence-base is because the implementation of educational interventions is usually poorly described and the effectiveness of individual methods is not evaluated. Thus, where studies find an effect, it is not possible to conclude which method or combination of methods is most effective.

Secondly, the intervention was delivered by staff employed by a leading UK TYA cancer charity. The presenters were highly motivated and skilled and can therefore be considered cancer awareness champions. While the style of delivery may vary by presenter, each presentation included the same core set of cancer messages. The significance of who delivers an intervention and the importance of champions has also been recognised in other school-based health promotion programmes [16].

### Strengths and limitations

To our knowledge, this is the first study in the UK to use the CAM to assess the effectiveness of a school-based educational intervention to raise cancer awareness. However, our study has several limitations. First, the sampling strategy may have introduced selection bias as students in schools with an existing relationship with Teenage Cancer Trust may be more receptive to cancer awareness messages due to teacher enthusiasm or reinforcement. Second, examination of change in cancer awareness by age was not possible in this study due to the younger age profile of the control school. Ethnicity could also not be examined because the small numbers of adolescents from other ethnic backgrounds, particularly in the control school (n=7), prevented meaningful comparison. The composition of the control school was a consequence of the sampling strategy which was selected pragmatically to facilitate both the first cross-sectional study of adolescents’ cancer awareness in the UK (reported elsewhere [25]) and the initial evaluation of the effectiveness of an existing educational intervention delivered by Teenage Cancer Trust to inform the development of future larger-scale school-based intervention studies. Third, the study did not assess differences in intervention effectiveness by socio-economic status. Fourth, the study did not analyse school-level variables to explain intervention effectiveness. Studies have shown that school ethos explains higher prevalence rates of smoking [27] and that working towards, or having, a Health Promoting School award explains mental well-being [17]. To address sampling limitations a larger study is required involving greater numbers of students and schools (without prior relationships with Teenage Cancer Trust). This would enable sub-group analyses by age and ethnicity. Future studies should also include measures of family affluence and school-level factors, such as school ethos and health education and promotion activity, to assess these as potential sources of variation in intervention effect. Intervention studies would be strengthened through the use of a cluster RCT design and multivariate analysis to examine the interplay between individual-, family-, and school-level variables, adjusted for clustering by school.

## Conclusions

Making a decision about whether to implement a school-based health promotion intervention should be based on evidence of effectiveness, cost and practicalities. This study shows that Teenage Cancer Trust presentations raised adolescent cancer awareness in the three schools where they were delivered. The intervention is provided free of charge to schools by Teenage Cancer Trust, does not require additional training of teaching staff and is delivered with minimal disruption to the school routine. Moreover, the topics covered during the presentation address key curriculum areas including health and well-being. Future studies should consider developing this intervention and evaluating the impact of the different methods used to raise adolescent cancer awareness and include individual-, family- and school-level explanatory variables.

## Endnotes

^1^ Rounding accounts for disparity between means at T_0_ and T_1_ reported to 1 decimal place (2.1) and reported figure for change (2.2).

## Abbreviations

CAM: Cancer awareness measure;NAEDI: National awareness and early diagnosis initiative;RCT: Randomised controlled trial;SD: Standard deviation;TYA: Teenage and young adult

## Competing interests

The authors declare that they have no competing interests.

## Authors’ contributions

RGK developed database and managed data entry, designed and conducted data analysis and interpretation, drafted and revised the manuscript. LF conducted data interpretation, drafted and revised the manuscript. PR provided statistical review of the manuscript. GH secured funding and ethics approval, managed data collection, conducted data interpretation, drafted and revised the manuscript. All authors read and approved the final manuscript.

## Pre-publication history

The pre-publication history for this paper can be accessed here:

http://www.biomedcentral.com/1471-2458/13/190/prepub
